# Development of a Magnetic Solid-Phase Extraction-Liquid Chromatography Targeted to Five Fluoroquinolones in Food Based on Aptamer Recognition

**DOI:** 10.3390/foods14050798

**Published:** 2025-02-26

**Authors:** Haiyan Zhou, Xiaofeng Yan, Yaning Song, Xiao Yang, Xianggui Chen, Yukun Huang

**Affiliations:** 1 Food Microbiology Key Laboratory of Sichuan Province, School of Food and Bioengineering, Xihua University, Chengdu 610039, China; 0120230102@mail.xhu.edu.cn (H.Z.); 13419195243@163.com (X.Y.); syn15208384079@163.com (Y.S.); 13076014204@163.com (X.Y.); chen_xianggui@mail.xhu.edu.cn (X.C.); 2 Chongqing Key Laboratory of Speciality Food Co-Built by Sichuan and Chongqing, Chengdu 610039, China

**Keywords:** fluoroquinolone, magnetic metal–organic framework, affinity recognition, food, detection

## Abstract

Fluoroquinolones (FQs) are present in trace amounts in the environment, from where they enter animal- and plant-derived food products. Long-term exposure to low-dose drugs poses a risk to human health and increases the pressure on antibiotic selection. Based on previous aptamer screening with high FQs specificity, this study combined a new aptamer recognition probe with a metal–organic framework (MOF) to obtain a sample pretreatment composite material with strong FQs specificity for multi-target analysis. Residual FQs were extracted from the complex food matrix via magnetic dispersive solid-phase extraction and examined using high-performance liquid chromatography. The method showed good linearity in a range of 0.39 to 200 µg/kg for five FQs in milk and fish samples, with a detection limit of 0.04–0.10 µg/kg and a quantitative limit of 0.13–0.33 µg/kg. This study successfully developed an effective sample pretreatment material and methodology for trace FQs identification in complex animal-derived food matrices.

## 1. Introduction

Although fluoroquinolones (FQs) are widely used to prevent and treat animal diseases due to their significant antibacterial properties, many challenges are associated with their residues because of improper use [1,2]. FQs can affect natural microbial communities and cause the formation of multi-drug-resistant bacteria [3], posing a risk to human and ecological health via the food chain [4]. Furthermore, FQs can disrupt human intestinal flora and cause chronic toxicity, including potential carcinogenicity, teratogenicity, and mutagenicity [5,6]. In recent years, the regulations regarding the use of FQ veterinary drugs have become clearer and various countries have established maximum residue limits (MRLs) for different animal-derived foods and animal parts [7,8,9]. China has set MRLs for the sum of enrofloxacin (ENR) and ciprofloxacin (CIP) in milk and fish at 100 µg/kg, lomefloxacin (LOW), pefloxacin (PEF), and ofloxacin (OFL) in animal-derived foods, including fish and honey, are 2 to 5 µg/kg [10,11]. FQs detection is challenging due to varying levels in food and mixed cross-medication, necessitating higher sensitivity requirements and multi-target high-throughput screening.

Among the quantitative methods of FQs, high-performance liquid chromatography (HPLC) is applied extensively [12,13]. The determination of trace FQs in complex matrices poses more requirements for sample pretreatment techniques, including accurate enrichment and rapid separation. Magnetic solid-phase extraction (MSPE) is a more common procedure because of its low cost, simplicity, and rapidity [14], although antibody-based pretreatment techniques have been developed [15]. Given the various forms of FQ pollution and the limited presence of these pollutants in actual situations, research focuses on improving the specificity, stability, and simple synthesis of adsorbents. Aptamers can fold into a stable three-dimensional structure (stem-loop, G-quadruplex, and pseudoknot) in certain conditions. These aptamers can then bind to small molecules via hydrogen bonding, electrostatic interaction, base accumulation forces, or hydrophobic interactions driven by enthalpy or entropy changes, offering benefits such as highly specific recognition and large-scale synthesis in vitro [16]. Recent aptamer-based methods for FQ detection have benefited from the advantages of aptamers, such as high sensitivity, good specificity, low cost, and rapid operation [17]. However, these aptamer-based biosensor detection methods darken the robust recognition capabilities of aptamers to multiple FQs, especially for simultaneous determination in complex food matrices. At present, aptamers for FQs are still ignored in the pretreatment of instrumental analysis, with only a few regarding tetracycline and bisphenol A aptamers [18,19], which all have some similarities to antibody-based pretreatment. Our group is continuously committed to research concerning the principles and applications of FQ aptamers with directed specificity. Currently, we have found that the aptamer W-G1 demonstrated excellent performance in targeting FQs after optimization during the collection of reported aptamers [20]. Therefore, this study aims to further develop an accurate and efficient sample pretreatment method for targeting multiple FQs in food using an enhanced aptamer with higher affinity, stability, and specificity for FQ recognition.

Metal–organic frameworks (MOFs) are porous materials with adjustable sizes and selectivity that have been widely applied in adsorption separation, energy storage, sensing, electrochemistry, and biomedicine [21,22]. MOFs’ pretreatment presents several challenges, such as poor stability, limited mechanical strength, and complex recovery processes. Therefore, combining MOFs with magnetic nanoparticles (MNPs) can enhance the development of composite materials more suitable for analytical determination [23,24,25,26]. MOFs are currently used for the environmental adsorption of dyes [27], heavy metal [3], tetracyclines [28], and FQs. Only a limited number of MOFs are used for detecting some FQs in food matrices [7,12,29,30], and more functional MOFs have been applied for the efficient removal of FQs [31,32,33,34,35]. Therefore, it is necessary to assess multi-target MOFs for FQ residue detection in food matrices. An MOF and covalent organic framework composite considerably improved the adsorption of FQs from water environments [36]. MOF-5, with good thermal stability, is a classical material that has been widely used since 1999 [37]. Magnetic MOF-5 presents rapid separation and recovery of targets. In addition, the combination of specific aptamers may provide a better balance between multi-target and specificity in determination.

Based on the above, the specific binding effect of the new aptamer recognition probe of FQs was combined with the non-specific adsorption of MOFs itself. A new aptamer-based magnetic solid-phase extraction (MSPE) adsorbent (Fe_3_O_4_/MOF-5-W-G1) was prepared and used to pretreat different animal-derived food products. In optimized conditions, the innovative dispersive-phase MSPE-liquid chromatography method was successfully used to analyze and determine five FQs in milk and fish samples.

## 2. Materials and Methods

### 2.1. Chemicals and Reagents

Enrofloxacin (ENR, ≥98%), ciprofloxacin (CIP, ≥98%), Lomefloxacin hydrochloride (LOW, ≥98%), pefloxacin (PEF, ≥98%) and ofloxacin (OFL, ≥99%) were obtained from Shanghai Yuanye Biological Co., Ltd. (Shanghai, China). Zinc acetate ((ZnCH_3_COO)_2_ · 2H_2_O), chemicals for phosphate buffer saline (PBS), and 2-aminoterephthalic acid (C_8_H_7_NO_4_) were all supplied by Shanghai Aladdin Chemical Co., Ltd. (Shanghai, China). N,N’-dimethylformamide (DMF) was acquired from Tianjin Zhiyuan Chemical Reagent Co., Ltd. (Tianjin, China). Glutaraldehyde aqueous solution (25% volume fraction), 1,6-hexanediamine (C_6_H_16_N_2_), and ferric chloride hexahydrate (FeCl_3_·6H_2_O) were obtained from Sinopharm Chemical Reagent Co., Ltd. (Shanghai, China). Triethylamine (C_6_H_15_N) was bought from Tianjin Jindong Tianzheng Fine Chemical Reagent Factory (Tianjin, China). Ethylene glycol was purchased from Chron Chemicals Co., Ltd. (Chengdu, China). ProElut polystyrene polymers (PLS) column (68,012) and ProElutQuE (64,535, special for FQs) were obtained from Dima Technology (Beijing, China). Ethanol, acetonitrile, acetic acid, n-hexane, and formic acid (HPLC-grade) were obtained from Macklin Biochemical Technology Co., Ltd. (Shanghai, China). Avidin and aptamer sequences (W-G1 and W) were acquired from Sangon Biotech (Shanghai, China) Co., Ltd. (Shanghai, China). All chemicals were analytical grade unless otherwise specified. All buffers were prepared with ultrapure water from a Milli Direct-Q water purification system (Millipore Corp., Bedford, MA, USA), including 140 mmol/L NaCl, 20 mmol/L Tris-HCl, 1 mmol/L MgCl_2_, 1 mmol/L CaCl_2_, pH 7.4.

### 2.2. Synthesis of Mixed Magnetic Materials

#### 2.2.1. Synthesis of Fe_3_O_4_-NH_2_

Ethylene glycol (30 mL), anhydrous sodium acetate (2 g), ferric chloride hexahydrate (1 g), and 1,6-hexanediamine (6.50 g) were added to a round-bottom flask, and the solution was heated to 50 °C by magnetic stirring in a water bath to make the solution appear as a wine red homogeneous colloid. The above solution was moved to the reactor, sealed, and continuously reacted at 198 °C for 6 h. After the reaction, the reactor was naturally cooled to room temperature, and Fe_3_O_4_-NH_2_ was obtained by magnetic separation using ultrapure water and anhydrous ethanol to wash the mixture several times. The above washed products were dried in a vacuum overnight at 65 °C and stored at room temperature.

#### 2.2.2. Synthesis of Fe_3_O_4_/MOF-5-NH_2_ Composite Magnetic Material

Refer to the method of Hu et al., 2013 [25], and synthesize after a slight improvement. The 2-aminoterephthalic acid (0.553 g, 3.05 mmol) was dissolved in 40 mL DMF. (ZnCH_3_COO)_2_ · 2H_2_O (1.70 g, 7.74 mmol) was dissolved in 50 mL of DMF. The zinc salt solution was dropped into the 2-aminobenzoic acid solution and stirred for more than 15 min to form a beige precipitate. Subsequently, 0.80 g of amino-functionalized Fe_3_O_4_ nanoparticles was immediately added under ultrasound and ultrasound for 30 min to make it evenly dispersed. The above solution was transferred to the reactor and sealed. After continuous high-temperature reaction at 120 °C for 24 h, the reactor was cooled to room temperature. The product was centrifuged, washed several times with DMF and ethanol, and dried overnight at 60 °C in a vacuum. The obtained brown-yellow product was sealed and stored at room temperature.

### 2.3. Assembly of Aptamer-Mixed Magnetic Materials

**Figure 1:**
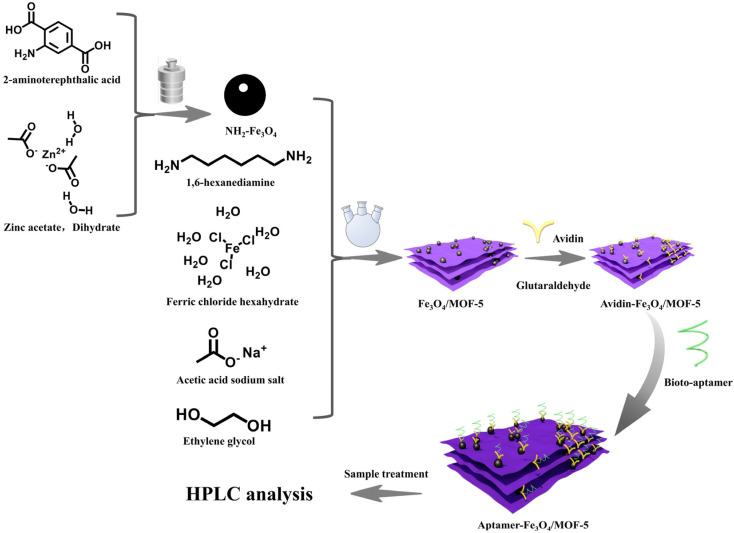
Schematic diagram of aptamer-mixed material assembly.

The assembly principle of the aptamer-mixed material in this experiment is shown in Figure 1.

The above-mentioned amino-modified magnetic material Fe_3_O_4_/MOF-5-NH_2_ (200 mg) was added to 20 mL of PBS (137 mmol/L NaCl, 207 mmol/L KCl, 10 mmol/L NaHPO_4_, 2 mmol/L KH_2_PO_4_). After ultrasonication to complete dispersion, 25 mL of glutaraldehyde aqueous solution (25:75, *v*/*v*) was added and incubated at 37 °C for 6 h at 120 r/min. The formed precipitate was retained by magnetic separation under an external magnet and washed with PBS several times. Finally, the precipitate was dispersed in 20 mL PBS solution (containing 1000 mL avidin) and incubated at 37 °C for 12 h at 120 r/min. The avidin-Fe_3_O_4_/MOF-5 was obtained by washing with PBS several times and was then suspended in 20 mL PBS and sealed at 4 °C. The above avidin-modified 200 mg avidin-Fe_3_O_4_/MOF-5 was dispersed in a 40 mL PBS solution containing 4 µL 100 µmol/L aptamer (W or W-G1), incubated at 110 r/min for 3 h at room temperature, PBS washed three times to remove non-specific binding aptamers to obtain aptamer-composite magnetic materials (W-Fe_3_O_4_/MOF-5 or W-G1- Fe_3_O_4_/MOF-5), and dispersed in 40 mL Tris-HCl, sealed at 4 °C.

### 2.4. Characterization of Materials

#### 2.4.1. Analysis of Fourier-Transform Infrared Spectroscopy (FTIR)

Infrared spectral analysis was carried out using a Thermo Fisher Scientific Nicolet 380 FTIR spectrometer (Waltham, MA, USA). The samples were prepared by the KBr pressing method. First, 1 mg of sample and 100 mg of KBr were weighed and then mixed in a mortar before grinding homogeneously and then pressed into a transparent sheet for testing. The measurement wavelength was 4000~400 cm^−1^ with a resolution of 4 cm^−1^.

#### 2.4.2. Determination of Zeta Potential

Pure water was used as the background, and materials were uniformly dispersed into the aqueous solution with the following parameter setting on the Malvern Zetasizer (Zen 3600): refractive index 1.33, laser wavelength 633 nm, and equilibration time 2 min. The zeta potential is closely related to the stability of the system, and the material is considered to have a more stable system when the absolute value of the zeta potential is greater than 30 mV.

#### 2.4.3. Material Microscopic Characterization

X-ray diffractometer (XRD) was used to study the crystallinity of the material (Bruker Corporation, Karlsruhe, Selb, Germany). The ground Fe_3_O_4_-NH_2_, MOF-5-NH_2_, and Fe_3_O_4_/MOF-5-NH_2_ samples were placed into the groove of the sample holder and flattened with a slide. The scanning angle was set to 5–70°, and the scanning speed was set to 4°/min. The morphology, structure, and properties of the materials were also characterized by a scanning electron microscope (SEM) (Hitachi, Tokyo, Japan). The material powder was ultrasonically dispersed in anhydrous ethanol before JEM 2100 Transmission Electron Microscope (TEM) analyses (JEOL Ltd., Tokyo, Japan).

#### 2.4.4. Other Characterization

For magnetic property characterization, materials were dispersed in ultrapure water, and a magnetic field was applied to observe their changes with and without the magnet. A Cary 100 Ultraviolet-visible Spectrophotometer (UV-vis) (Varian Corporation, Palo Alto, CA, USA) was used to measure the UV absorbance changes before and after avidin and aptamer modification for the characterization of Fe_3_O_4_/MOF-5-W-G1 composites.

### 2.5. Sample Pretreatment

The FQs solution was added to 1 g of milk, diluted to 6, 47, and 100 µg/kg, respectively, and transferred to a centrifuge tube. Then, 9 mL ethanol was added, mixed thoroughly, and centrifuged at 5000 r/min for 10 min. The sample solution was incubated with 200 mg of the aptamer-mixed magnetic material at 110 r/min for 60 min, after which the supernatant was discarded via magnetic separation. The ethanol/acetic acid (97:3, *v*/*v*) was eluted until no target substance remained in the eluent, which was collected, heated at 45 °C, and dried using nitrogen. The sample was redissolved in 1 mL methanol and filtered through a membrane before HPLC analysis.

A 1 g fish sample was placed in a 50 mL centrifuge tube. Then, 1 mL of the FQs solution was added at the above respective concentrations, extracted with 3 mL of formic acid/acetonitrile (2:98, *v*/*v*), and homogenized for 30 s. The extract was centrifuged at 5000 r/min for 5 min, after which the supernatant was transferred to the colorimetric tube. These steps were repeated 3 times, and the subsequent extracts were combined. The extract was diluted to 10 mL with formic acid-acetonitrile (2:98, *v*/*v*) and degreased with 10 mL acetonitrile saturated n-hexane. After discarding the n-hexane layer, the lower solution was filtered through a membrane and incubated with 200 mg of the mixed magnetic material aptamer at 110 r/min for 60 min. The elution procedure was the same as that used for the milk sample treatment.

### 2.6. Optimization and Application of HPLC-Fe_3_O_4_/MOF-5-W-G1 Detection Method

#### 2.6.1. Liquid Chromatography Conditions

The chromatographic conditions were referred to as the national standard conditions [38,39]. The instrumental analysis was carried out using an HPLC system (Waters Corporation Milford, CT, USA), which was equipped with the model E2695 HPLC pump and the model 2489 UV detector. Mobile phase A was the 0.34% phosphoric acid aqueous solution and adjusted to pH 2.50 with triethylamine, while mobile phase B comprised acetonitrile. The isocratic elution program with 85% mobile phase A and 15% mobile phase B was performed at a flow rate of 0.8 mL/min. An Ultimate XB-C_18_ column (4.60 mm × 25 cm, 5 µm, Welch Technology Corporation, Shanghai, China) was kept at 30 °C for separation. The detection wavelength was set to 275 nm, and the injection volume was 10 µL.

#### 2.6.2. Establishment of Standard Curve

The single-product standard stock solution was prepared by dissolving 10 mg of five FQs standards in 1 mL methanol. The mixture was diluted to 10 mL and stored at −20 °C. The mixed standard working solution was prepared by gradually diluting the standard stock solution to 200, 100, 50, 25, 12.50, 6.25, 3.13, 1.56, 0.78, and 0.39 µg/kg. The storage conditions were the same as above. A calibration plot was obtained by using the peak area (AU) of FQs as the ordinate and their corresponding concentration levels as the abscissa. Matrix Effects (ME) were evaluated by analyzing the slopes of standard curves diluted with matrix solution after purification and organic extractant, respectively. It can be calculated by Equation (1), where K_a_ (matrices) and K_b_ (organic solvent) are the slopes of curves.

(1) $$ME\left(\%\right)=\left(\frac{Ka}{Kb}-1\right)\times 100$$

#### 2.6.3. Optimization of Absorption Conditions

The amount of the assembled materials in the pretreatment process was optimized. The aptamer-mixed magnetic material of 50 mg, 100 mg, 150 mg, 200 mg, and 250 mg were selected for single-target ENR (0.01 mg) adsorption, and then successively shaken at 110 r/min for 60 min. The supernatants were detected after the sorbents had been separated. Similarly, 200 mg aptamer-mixed magnetic material and ENR were examined at 110 r/min for 30 min, 40 min, 50 min, 60 min, 70 min, and 80 min to obtain the optimal extraction reaction conditions. The adsorption capacity was calculated by Equation (2):

(2) $$Adsorption\;capacity=\frac{A_{0}-A}{A_{0}}$$

where *A* is the intensity of the supernatants after MSPE pretreatment, $A_{0}$ is the initial intensity before MSPE pretreatment.

#### 2.6.4. Method Evaluation

Milk and fish samples were taken from nearby supermarkets. The fish were homogenized prior to the experiment. The coefficient of determination (R^2^), limits of detection (LODs), limits of quantitation (LOQs), accuracy, and precision were evaluated to validate the applicability of this method [7,12,27]. The mixture of five FQs was added to the sample (fish and milk) with three levels of spiked concentration (6, 47, and 100 µg/kg). The pretreatment operation was carried out according to Section 2.5 under the previous optimized experimental reaction conditions and detected as described in Section 2.6.1. The accuracy was evaluated by the recoveries from triplicate following Equation (3):

(3) $$Recovery\left(\%\right)=\frac{C-B}{C_{0}}\times 100$$

where *C* is the real detection concentration for FQs-spiked samples, *B* is the actual detection concentration for the non-spiked samples, and $C_{0}$ is the ideal added concentration. Precision is reflected by relative standard deviation (RSD). Specifically, the RSD analyses were assessed by using the above method to detect FQs (100 µg/kg) fortified samples in six replicates on the same day.

## 3. Results and Discussion

### 3.1. Characterization of Fe_3_O_4_/MOF-5-NH_2_ Material

#### 3.1.1. Fourier-Transform Infrared Spectroscopic Characterization

**Figure 2:**
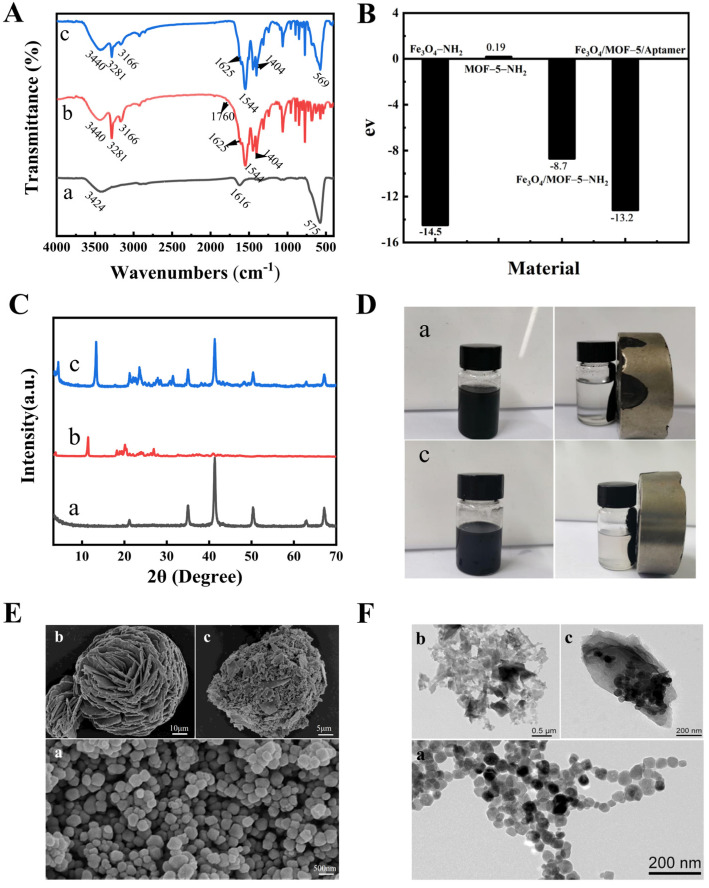
Material characterization results: (**A**) Fourier-transform infrared spectroscopy; (**B**) Zeta Potential; (**C**) X-ray diffraction (XRD); (**D**) Magnetic property characterization; (**E**) SEM; (**F**) TEM; a, b, and c, represent Fe_3_O_4_-NH_2_, MOF-5-NH_2_, and Fe_3_O_4_/MOF-5-NH_2_, respectively.

As shown in Figure 2A, the characteristic Fe-O peak at 575 cm^−1^, the amino N-H bending vibration peak at 1616 cm^−1^, the N-H stretching vibration of the amino group at 3424 cm^−1^, and the O-H stretching vibration of water in Fe_3_O_4_-NH_2_ indicated the successful Fe_3_O_4_-NH_2_ synthesis. The appearance of -COOH at 1760 cm^−1^ in MOF-5-NH_2_ indicated the presence of free -COOH on the MOF-5-NH_2_ surface. The C=O band I at 1625 cm^−1^, the N-H in-plane bending vibration band II at 1544 cm^−1^, and the C-N stretching vibration band III at 1404 cm^−1^ in Fe_3_O_4_/MOF-5-NH_2_ indicated amide group formation. Furthermore, the disappearance of -COOH and the appearance of amide groups in Fe_3_O_4_/MOF-5-NH_2_ indicated that Fe_3_O_4_/MOF-5-NH_2_ synthesis was based on the reaction between -COOH and -NH_2_ on the MOF-5-NH_2_ and Fe_3_O_4_-NH_2_ surfaces. In addition, the presence of amide groups in MOF-5-NH_2_ can be attributed to the inherent -COOH and -NH_2_ functional groups in the 2-aminoterephthalic acid molecule. In high-temperature conditions, a portion of these functional groups may undergo binding to cause amide formation. In addition, the characteristic Fe-O peak at 569 cm^−1^ further indicated successful Fe_3_O_4_/MOF-5-NH_2_ synthesis.

#### 3.1.2. Zeta Potential Characterization

The material synthesis was characterized by analyzing the differences between the zeta potential values. A significant change was evident in the potential value after combining Fe_3_O_4_-NH_2_ and MOF-5-NH_2_ (Figure 2B), indicating that Fe_3_O_4_-NH_2_ and MOF-5-NH_2_ had successfully synthesized Fe_3_O_4_/MOF-5-NH_2_. In addition, the potential value decreased by 4.50 eV after the Fe_3_O_4_/MOF-5-NH_2_ mixed magnetic material was combined with the aptamer, indicating that the negative charge of the aptamer affected the surface charge of the mixed material, indicating successful incorporation.

#### 3.1.3. X-Ray Diffraction Characterization

XRD was used to characterize the impact of Fe_3_O_4_-NH_2_ on the porous MOF-5-NH_2_ structure. The small angle peaks (2θ = 21.23, 35.08, 41.33, 50.42, 62.86, and 67.27) of Fe_3_O_4_-NH_2_ corresponded with the Fe_3_O_4_ structure in the 99-0073 standard card (Figure 2C). MOF-5-NH_2_ exhibited a similar structural pattern to that previously reported for MOF via XRD [40,41]. Furthermore, Fe_3_O_4_-NH_2_ addition did not significantly affect the MOF-5-NH_2_ structural integrity.

#### 3.1.4. Magnetic Property Characterization

A comparison was performed to confirm that Fe_3_O_4_-NH_2_ did not affect the magnetic properties of mixed Fe_3_O_4_/MOF-5-NH_2_ magnetic materials after adding MOF-5-NH_2_. Figure 2D illustrates the dispersion of Fe_3_O_4_-NH_2_ and Fe_3_O_4_/MOF-5-NH_2_ before the magnet adsorption process in ultrapure water. The experimental results showed that Fe_3_O_4_-NH_2_ and Fe_3_O_4_/MOF-5-NH_2_ exhibited uniform distribution in ultrapure water. When the applied magnetic field was near the magnetic material, Fe_3_O_4_-NH_2_ and Fe_3_O_4_/MOF-5-NH_2_ responded rapidly and aggregated in the direction of the magnet, which indicated that both materials displayed good magnetic properties.

#### 3.1.5. SEM and TEM Characterization

SEM (Figure 2E) and TEM (Figure 2F) were used to characterize the morphological structure of the synthesized hybrid magnetic MOF-5. SEM showed that the Fe_3_O_4_-NH_2_ nanoparticles displayed an irregular, spherical morphology, while MOF-5-NH_2_ exhibited uneven lamellar clustering. Fe_3_O_4_/MOF-5-NH_2_ showed a combination of these morphologies, while the MOF-5 surface was modified with irregular spherical Fe_3_O_4_ nanoparticles. The TEM results regarding the morphology of Fe_3_O_4_-NH_2_, MOF-5-NH_2_, and Fe_3_O_4_/MOF-5-NH_2_ were consistent with the SEM findings. The results showed successful MOF-5 magnetization by the Fe_3_O_4_ nanoparticles and Fe_3_O_4_/MOF-5-NH_2_ mixed magnetic material synthesis.

### 3.2. Characterization of Fe_3_O_4_/MOF-5-W-G1 Composites

**Figure 3:**
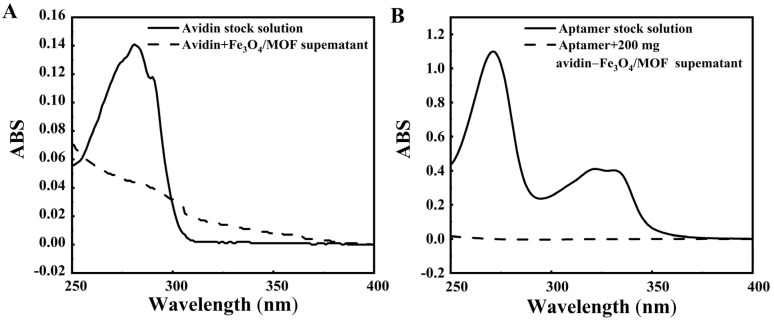
The UV absorption spectra. (**A**) The avidin-magnetic material; (**B**) The aptamer-magnetic material.

To successfully construct the Fe_3_O_4_/MOF-5-aptamer, glutaraldehyde was used to covalently link avidin and the amino groups on the Fe_3_O_4_ surface. This process facilitated the attachment of avidin to the Fe_3_O_4_ surface, after which the biotin–avidin system was used to further connect aptamer (W-G1). Therefore, the UV absorbance changes before and after avidin and aptamer modification were measured at 280 nm and 260 nm, respectively, to characterize the relationship between the avidin and the amino groups on the Fe_3_O_4_ surface and the successful connection of the avidin–biotin system to the Fe_3_O_4_/MOF-5-aptamer. Significant changes were evident before and after the avidin modification of the mixed magnetic material, indicating that avidin successfully altered Fe_3_O_4_/MOF-5 (Figure 3A). The UV absorption of the aptamer decreased significantly before and after combining the biotin-aptamer with the avidin-modified mixed magnetic material, indicating a successful Fe_3_O_4_/MOF-5-aptamer (W-G1) assembly (Figure 3B).

### 3.3. Optimization of the Sample Pretreatment Adsorption Time

**Figure 4:**
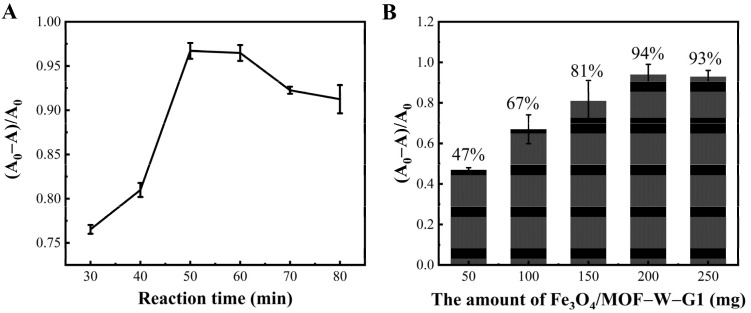
The reaction optimization: (**A**) Optimization of the mixed magnetic material-aptamer and ENR quantities; (**B**) Optimization of the mixed magnetic material-aptamer and ENR reaction times.

To optimize the Fe_3_O_4_/MOF-5-W-G1 reaction time, 200 mg Fe_3_O_4_/MOF-5-W-G1 was used to adsorb 1 mL ENR at a concentration of 0.01 mg/mL. The results showed that the capacity of Fe_3_O_4_/MOF-5-W-G1 to adsorb to ENR increased gradually as the incubation time was extended. A strong adsorption capacity for the target substance was evident at an incubation time of 50 min (Figure 4A), with an adsorption rate of 96%. The FQs adsorption performance decreased as the incubation time increased. This could be attributed to the porous structure of the MOF-5 material, which might cause FQs to bind to the non-specific recognition sites of the adsorption material. As the incubation time increased, the binding force of the FQs gradually decreased, decreasing their adsorption performance.

### 3.4. Optimization of Extractant Dosage

To enhance the adsorption efficiency of Fe_3_O_4_/MOF-5-W-G1 for FQs veterinary drug targets, this experiment selected different concentrations for single-target ENR adsorption. Figure 4B shows the experimental results. The adsorption effect of the adsorbent on 1 mL ENR (10 µg/kg) gradually increased as the adsorbent concentration increased, with the ENR adsorption efficiency reaching 94% at 200 mg. Therefore, 200 mg of Fe_3_O_4_/MOF-5-W-G1 was selected to pretreat veterinary drugs containing FQs.

### 3.5. Verification of the Fe_3_O_4_/MOF-5-W-G1 Adsorption Performance

**Figure 5:**
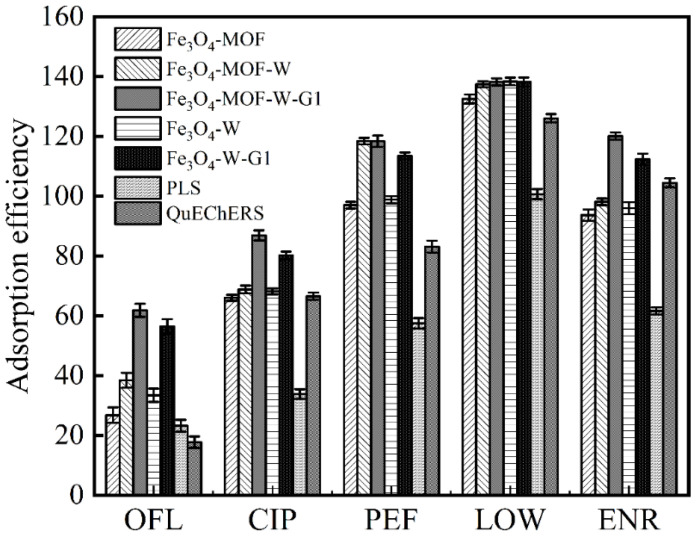
A comparison between the adsorption efficiency of the adsorbent materials.

For evaluating the adsorption effect of the adsorption materials on five FQs in this experiment, we used the same instrument conditions to determine the sample recovery rate of all MSPE materials (including Fe_3_O_4_-MOF-5, Fe_3_O_4_-MOF-5-W, Fe_3_O_4_-MOF-5-W-G1, Fe_3_O_4_-W, Fe_3_O_4_-W-G1): commercial PLS column and commercial QuEChERs extraction packages that can be used to adsorb FQs in this experiment. Compared with Fe_3_O_4_-MOF-5-W, Fe_3_O_4_-W, and Fe_3_O_4_-W-G1, the adsorption effect of the Fe_3_O_4_-linked aptamer was stronger than that of Fe_3_O_4_-MOF-5 (Figure 5), which indicated that the targeted aptamer adsorption to the FQs was higher than MOF-5. The Fe_3_O_4_-MOF-5 exhibited a notably lower adsorption capacity to the five FQs than Fe_3_O_4_-MOF-5-W and Fe_3_O_4_-MOF-5-W-G1, indicating that the aptamer was connected, further enhancing its adsorption efficacy. The adsorption effect of Fe_3_O_4_-MOF-5-W-G1 was superior to that of Fe_3_O_4_-MOF-5-W, demonstrating that the affinity of W-G1 was higher than that of the original W. The results showed that Fe_3_O_4_-MOF-5-W-G1 displayed the best adsorption effect on the five FQs. Obviously, based on the specific adsorption of materials, the application of aptamers with greater affinity improves their specific binding ability to some extent, which can accurately identify and bind targets in complex matrices for achieving efficient extraction-purification pretreatment.

### 3.6. Methodological Evaluation

The standard liquid chromatograms of FQs are shown in Figure S1, while the correlation coefficient (R), detection limit, and quantitative limit are summarized in Table 1. The method showed good linearity (R > 0.9985) in a range of 0.39 to 200 µg/kg for five FQs, with a detection limit of 0.04–0.10 µg/kg and a quantitative limit of 0.13–0.33 µg/kg. The spiked recovery chromatogram (Figure S2) showed that this method successfully separated several FQs at low, medium, and high spiked concentrations in actual samples. After pretreatment with Fe_3_O_4_-MOF-5-W-G1 composites, ME of the five FQs in fish samples can be accepted (3.37% ≤ |ME| ≤ 17.47%). In addition to OFL, other absolute values of ME in milk were also below 20%, which showed that the proposed method was feasible for the detection and analysis of FQs in fish or milk matrix (Table 1). Compared with other detection methods for FQs [7,12,13,15,29], the proposed method in this work presents certain advantages in detection cost, detection and quantitative limits. Specifically, the accurate trace detection of five FQs in milk and fish was achieved without the use of commercial antibody-based immunoaffinity microextraction while meeting the lowest LODs and LOQs (Table S1).

According to the optimized experimental conditions, the new Fe_3_O_4_-MOF-5-W-G1 extractant with the best sample recovery effect was used to determine the actual sample and the accuracy. Table 2 shows the experimental results. Except for OFL, the recovery rates of the other FQs were 84.71% to 102.06%, indicating that the Fe_3_O_4_-MOF-5-W-G1 material exhibited good adsorption ability for CIP, PEF, LOW, and ENR in milk and fish samples. The adsorption effect of low-concentration OFL was poor, and the recovery rate was 65.34–71.50%. This is possibly related to OFL hydrophobicity. In addition, the RSD values of this method in the spiked samples ranged from 1.02% to 2.53%, indicating excellent detection accuracy. According to the recoveries, this method needs to be improved compared with other methods (Table S1). In addition, the results of RSD in Table 1 all also below 3%, which indicates elegant precision.

In short, the FQs detection method established in this paper displayed an excellent linear range, detection limit, and quantitative limit (Table S1). In addition, the new aptamer recognition probe exhibited high specificity for a variety of FQs at a low cost. The Fe_3_O_4_ and MOF-5 combination enabled adsorbent separation from the sample matrix via an external magnet, which facilitated the capture of trace FQ residues in food matrices. Therefore, Fe_3_O_4_/MOF-5-W-G1 is an ideal adsorbent for pretreatment during FQ detection in animal-derived food products.

## 4. Conclusions

Based on a broad-spectrum short-chain FQs aptamer of 40-nt long obtained in previous research by the team, this study establishes a modified recognition-probe-functionalized composite magnetic material (Fe_3_O_4_-MOF-5-W-G1) for MSPE pretreatment in food samples. This provides a simple method to remove the adsorbent from the matrix without additional centrifugation or filtration, allowing the measured components to be rapidly separated from the sample matrix using an external magnetic field. The new aptamer-MSPE extractant displays good thermal stability and chemical resistance, as well as high selectivity and recognition specificity. When the type of aptamer is changed, the method can also be used to detect other drugs or FQs residues. Therefore, this study encompasses both theoretical design and practical application, offering valuable technical support for the efficient and accurate identification of various FQs veterinary drug residues in food. It is worth noting that the low-concentration recovery for OFL with highly polar will need to be further improved.

## Figures and Tables

**Table 1:** Retention time, linearity, LODs, LOQs, RSD, and MEs of the proposed method.

Target	Retention Time	Correlation Coefficient	Linear Range (µg/kg)	LODs (µg/kg)	LOQs (µg/kg)	RSD (%, *n* = 6)	ME (Fish, %)	ME (Milk, %)
OFL	13.46	0.9988	0.39–200	0.04	0.15	2.01	17.47	23.12
CIP	14.77	0.9989	0.39–200	0.05	0.17	1.89	−6.79	5.14
PEF	15.37	0.9998	0.39–200	0.07	0.23	1.90	6.32	2.50
LOW	17.95	0.9995	0.39–200	0.10	0.33	1.86	−3.37	13.45
ENR	22.57	0.9998	0.39–200	0.04	0.13	2.21	6.52	−11.19

**Table 2:** Determination of recoveries and concentrations of FQs in spiked samples at three levels.

Matrices		Milk			Fish		
Target	Added (µg/kg)	Measured Quantity (µg/kg)	Recoveries (%)	RSD (*n* = 3, %)	Measured Quantity (µg/kg)	Recoveries (%)	RSD (*n* = 3, %)
OFL	6	4.29	71.50	2.13	3.92	65.34	2.35
	47	40.78	86.77	1.23	37.42	79.63	1.67
	100	99.82	99.82	2.33	88.76	88.76	2.01
CIP	6	5.22	86.99	2.31	5.29	88.12	2.12
	47	44.03	93.68	1.40	45.68	97.19	1.87
	100	93.64	93.64	1.12	102.05	102.05	1.65
PEF	6	5.08	84.71	1.02	5.82	97.08	2.45
	47	44.94	95.62	2.20	47.51	101.09	1.25
	100	101.11	101.11	2.08	93.36	93.36	1.03
LOW	6	5.43	90.52	2.44	5.51	91.75	2.13
	47	44.89	95.51	1.21	43.66	92.90	2.53
	100	99.29	99.29	2.21	95.93	95.93	2.15
ENR	6	5.40	90.07	2.11	5.49	91.54	2.31
	47	45.52	96.63	1.11	45.73	97.30	1.39
	100	102.06	102.06	2.03	91.84	91.84	1.21

## Data Availability

Data are contained within the article/Supplementary Materials.

## Supplementary Materials

The following supporting information can be downloaded at: https://www.mdpi.com/article/10.3390/foods14050798/s1, Figure S1: Standard liquid chromatograms of FQs at a concentration of 200 µg/kg; Figure S2: Chromatogram (A) the spiked fish sample recovery; (B) the spiked milk sample; a, b and c represent the chromatograms when the sample is spiked FQs at 6, 47 and 100 µg/kg, respectively; Table S1: Comparison with other detection methods for FQs.
