# Supplementary material for: Development of a Magnetic Solid-Phase Extraction-Liquid Chromatography Targeted to Five Fluoroquinolones in Food Based on Aptamer Recognition
Source: Foods. 2025 Feb 26;14(5):798. doi: 10.3390/foods14050798 (PMC11899132; doi:10.3390/foods14050798)
Supplement: Supplementary file 1 [file foods-14-00798-s001.zip › foods-3476137-supplementary.pdf]

foods

Supplementary materials

Development of a Magnetic Solid-Phase Extraction-Liquid Chromatography Targeted to Five Fluoroquinolones in Food Based on Aptamer Recognition

Haiyan Zhou [1,†], Xiaofeng Yan [1,†], Yaning Song [1], Xiao Yang [1,2], Xianggui Chen [1,2] and Yukun Huang [1,2,*]

[1] Food Microbiology Key Laboratory of Sichuan Province, School of Food and Bioengineering, Xihua University, Chengdu 610039, China; 0120230102@mail.xhu.edu.cn (H.Z.); 13419195243@163.com (X.Y.); syn15208384079@163.com (Y.S.); 13076014204@163.com (X.Y.); chen_xianggui@mail.xhu.edu.cn (X.C.)
[2] Chongqing Key Laboratory of Speciality Food Co-Built by Sichuan and Chongqing, Chengdu 610039, China
* Correspondence: huangyukun@xhu.edu.cn; Tel.: +86-02887720552
† These authors contributed equally to this work.

Table S1. Comparison with other detection methods for FQs.

Matrix	Adsorbent	Method	Target	LODs (μg/kg)	LOQs (μg/kg)	Recoveries (%)	Reference
Breast milk	/	HPLC-FD	MOX	300	1000	99.10-100.64	[13]
Milk	IA-MEPS	HPLC-FD	ENR, CIP, LOM, OFL, NOR, FLE, DAN, ORB	0.05-0.1	0.15-0.3	53.9-90.6	[15]
Milk, river water	RAM-MIPs	HPLC–UV	ENR, PEF, OFL, NOR, GAT	0.93–3.15	3.15-10.52	80.7–105.9	[27]
Milk	FPy-COF@ PDA@Fe_3O_4	HPLC-UV	ENR, CIP, LOM, NOR, DFX, MAR	2.3-6.8	7.5-22.7	77.8-110.4	[12]
Milk	Fe_3O_4@MOF @PLS	HPLC-FD	ENR, CIP, NOR	0.21-1.33	0.71-4.42	95.4-110.3	[7]
Milk, fish	Fe_3O_4/MOF-5-W-G1	HPLC-UV	ENR, CIP, LOM, PEF, OFL	0.04-0.10	0.13-0.33	65.34-102.06	This work

Note: Moxifloxacin (MOX), norfloxacin (NOR), ofloxacin (OFL), fleroxacin (FLE), ciprofloxacin (CIP), danofloxacin (DAN), lomefloxacin (LOM), enrofloxacin (ENR), orbifloxacin (ORB), pefloxacin (PEF), gatifloxacin (GAT), marbofloxacin (MAR), difloxacin (DFX); immunoaffinity microextraction in a packed syringe (IA-MEPS); restricted access media-imprinted nanomaterials based on a metal organic framework (RAM-MIPs); fluorinated porphyrin-based covalent organic frameworks (FPy-COF), polydopamine (PDA); ProElut polystyrene polymers (PLS); W-G1 means aptamer sequences.

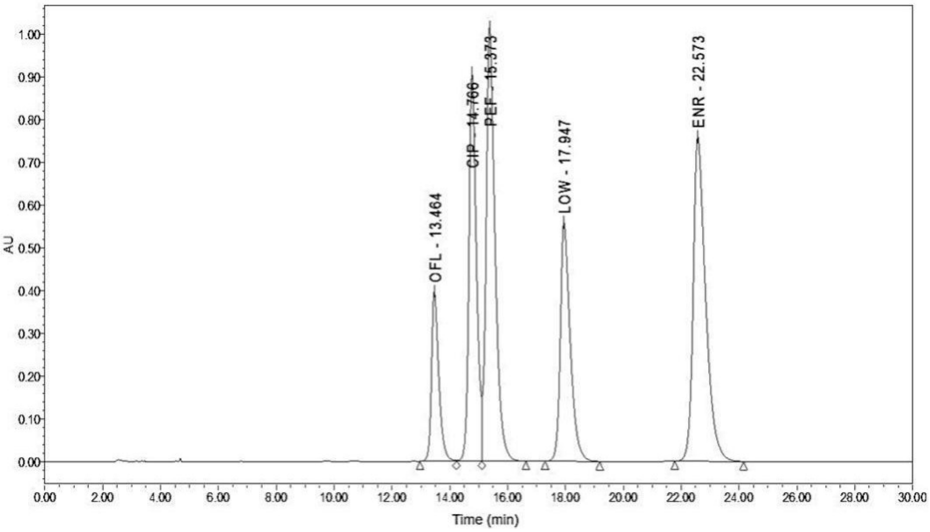

Figure S1. Standard liquid chromatograms of FQs at a concentration of 200 μg/kg

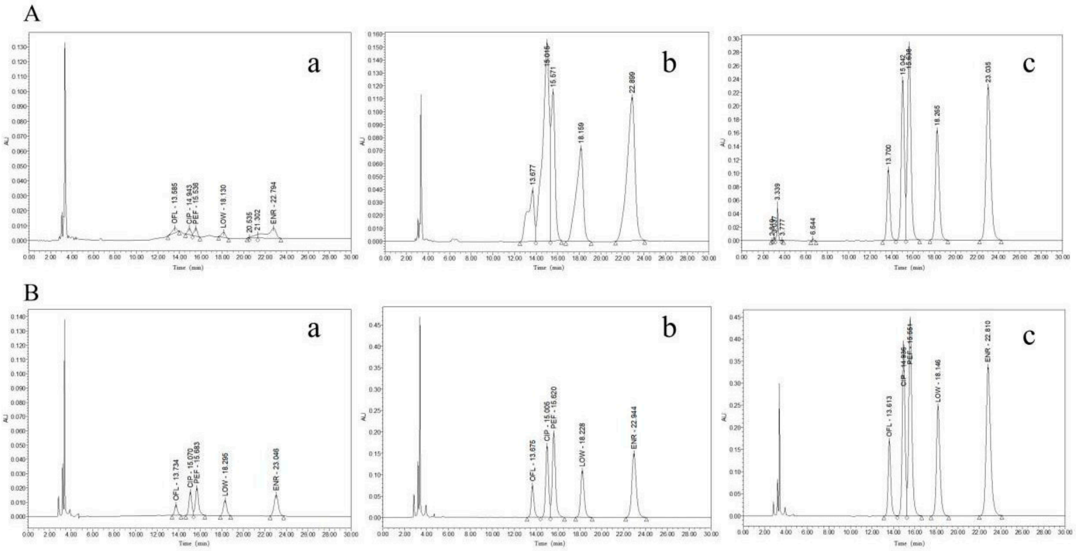

Figure S2. Chromatogram (A) the spiked fish sample recovery; (B) the spiked milk sample; a, b and c represent the chromatograms when the sample is spiked FQs at 6, 47 and 100 μg/kg, respectively.
